# Early fusion outcome after surgical treatment of single-level and multi-level pyogenic spondylodiscitis: experience at a level 1 center for spinal surgery—a single center cohort study

**DOI:** 10.1186/s13018-023-03584-0

**Published:** 2023-02-15

**Authors:** Motaz Hamed, Simon Brandecker, Tim Lampmann, Harun Asoglu, Abdallah Salemdawod, Erdem Güresir, Hartmut Vatter, Mohammed Banat

**Affiliations:** 1 Department of Neurosurgery, University Hospital of Bonn, Venusberg-Campus 1, Building 81, 53127 Bonn, Germany; 2 Center for Advanced Imaging Research, Department of Diagnostic Radiology and Nuclear Medicine, University of Maryland Marlene and Stewart Greenebaum, Comprehensive Cancer, Center University of Maryland, Baltimore, USA

**Keywords:** Urgent spinal surgery, Spinal instrumentation, Pyogenic spondylodiscitis, Single-level, Multi-level, Intervertebral fusion and instrumentation

## Abstract

**Study design:**

Retrospective single center cohort study.

**Purpose:**

Spinal instrumentation in combination with antibiotic therapy is a treatment option for acute or chronic pyogenic spondylodiscitis (PSD). This study compares the early fusion outcome for multi-level and single-level PSD after urgent surgical treatment with interbody fusion in combination with fixation.

**Methods:**

This is a retrospective cohort study. Over a 10 year period at a single institution, all surgically treated patients received surgical debridement, fusion und fixation of the spine to treat PSD. Multi-level cases were either adjacent to each other on the spine or distant. Fusion rates were assessed at 3 and 12 months after surgery. We analyzed demographic data, ASA status, duration of surgery, location and length of spine affected, Charlson comorbidity index (CCI), and early complications.

**Results:**

A total of 172 patients were included. Of these, 114 patients suffered from single-level and 58 from multi-level PSD. The most frequent location was the lumbar spine (54.0%) followed by the thoracic spine (18.0%). The PSD was adjacent in 19.0% and distant in 81.0% of multi-level cases. Fusion rates at the 3 month follow-up did not differ among the multi-level group (*p* = 0.27 for both adjacent and distant sites). In the single-level group, sufficient fusion was achieved in 70.2% of cases. Pathogen identification was possible 58.5% of the time.

**Conclusions:**

Surgical treatment of multi-level PSD is a safe option. Our study demonstrates that there was no significant difference in early fusion outcomes between single-level and multi-level PSD, whether adjacent or distant.

## Introduction

Pyogenic spondylodiscitis (PSD) is a known acute or chronic complication of primary pyogenic infection [1]. However, it can also develop as a complication of surgical or invasive spinal procedures [2]. PSD can be treated conservatively or surgically [3, 4], and can affect a single level or multiple levels. Patients benefit from early surgical treatment of PSD and spinal infection [5].

The urgent surgical treatment of PSD, whether single-level or multi-level, consists of spinal instrumentation through interbody fusion and fixation in combination with antibiotic therapy [6–9]. While there are data for treatment outcomes concerning single-level PSD, data on outcomes for multi-level; PSD after urgent surgical treatment are scarce.

Generally, a common typical early complication after fixation and instrumentation of the spine addressed by various authors is non-fusion with related material failure, with a range from 1–15% up to 63% [10–12]. Several factors affect bone fusion, such as bone quality, patient age, and sex [13]. The choice of fusion materials in the spine, such as PEEK cages or other cage types such as titanium, affects both fusion quality/time and the procedure used [14]. Despite this, recommendations on fusion procedure in relation to several variables such as clinical parameters have not been made in the literature.

This study evaluates potential differences in early fusion rates in patients treated surgically for single-level and multi-level PSD.

## Methods

### Patient selection and inclusion criteria

In this retrospective single center cohort study, data on all the patients treated at our center for PSD between January 2006 and December 2016 and aged ≥ 18 years were analyzed. We analyzed demographic data, ASA status, duration of surgery, localization and length of spine affected, Charlson comorbidity index (CCI) [15], and early complications.

The inclusion criterion was pyogenic spondylodiscitis (PSD). The indications for urgent surgery were systemic infection and instability with destruction of bone, with or without neurological symptoms. PSD was diagnosed on the basis of the preoperative MRI (including gadolinium-enhanced T1-weighted MR sequences) and CT scans in combination with preoperative systemic inflammatory laboratory parameters (white blood cell count, serum C-reactive protein). Laboratory investigation of inflammatory markers was performed in our center as previously described [16]. Additionally, we recorded clinical signs of PSD such as lumbar axial pain and abnormal body temperature. *Blood cultures were taken preoperatively from all patients, and microbiological swabs were taken intraoperatively. In addition to microbiological cultures, histological slides were sent to the Department of Pathology. We included patients here who had evidence of chronic granulomatous inflammation in the slides. Microbiological detection of germs was also considered an inclusion criterion.*

**Fig. 1:**
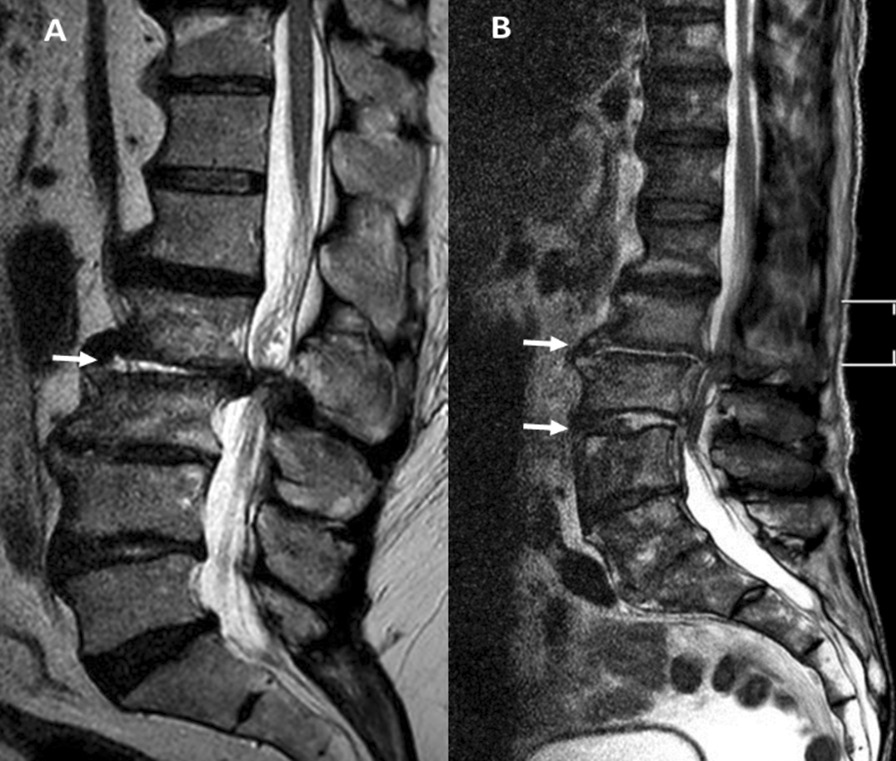
MRI STIR sagittal sequence: SL PSD (**A**) and adjacent ML PSD (**B**)

A radiological image of the spine was produced prior to surgery by CT and T2 sequence MRI scans to show edema at the disc and at the bone (Fig. 1) [17, 18].

The surgical approach was determined by the location and length of spine affected. The follow-up evaluation was based on the results of CT scans and of laboratory and microbiological tests. Early fusion was assessed using CT scans at 3 and 12 months after surgery. Early postoperative complications were assessed using a publicly available list of events introduced by the Agency for Healthcare Research and Quality and the Center for Medicare and Medicaid Services and referred to as patient safety indicators (PSIs) and hospital-acquired conditions (HACs) [19–21].

*Exclusion criteria* Patients with tumors, degenerative or activated osteochorndrosis, or other pathologies were excluded. In addition, patients who were histopathological clear without evidence of infection, but osteochorndrosis or degenerative advanced disc disease were also excluded later.

### Surgical procedure

**Fig. 2:**
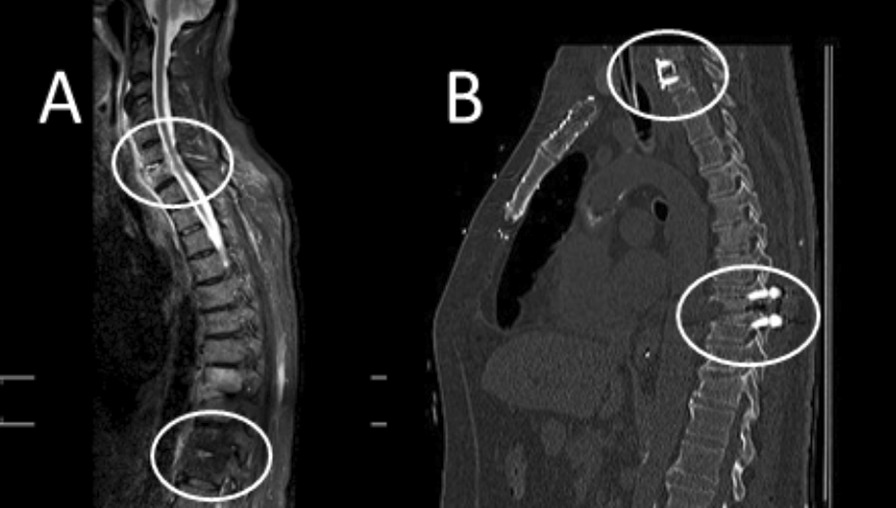
MRI image showing ML PSD at cervical und thoracic spine, **A** before surgery, **B** after surgery (postoperative CT scan)

The surgical procedure generally involved anesthesia. The primary goals of the operation were debridement of infection and stability of the spine. Our standard surgical procedure consists of the following steps for the lumbar and thoracic spine: open transpedicular screw implantation, spinal canal decompression via laminectomy, debridement of the infections, removal of the infected disc, then PEEK cage and bone implantation for fusion (Fig. 2, A: Pre surgery distant PSD; B: after surgery, lower circles).

Primary treatment of PSD of the cervical spine consists of anterior cervical discectomy and fusion (ACDF) with anterior plate fixation (Fig. 2, A: pre surgery distant PSD; B: after surgery, upper circles). To eliminate as much as possible bias due to the skill or experience of the surgeon, operations were carried out by only four neurosurgeons in the center.

All of the patients received systemic antibiotic therapy intravenously for 2 weeks, then orally for 10 weeks. Patients underwent early postoperative mobilization with physiotherapy. Postoperatively, CT scans were performed immediately after surgery to reconfirm accurate decompression and screw implantation. Further clinical and imaging follow-up using CT scans were performed at 3 and 12 months after surgery.

### Radiological evaluation

Postoperative imaging data were analyzed by an independent neuroradiologist in accordance with the center’s standards. Abnormalities around the screws and signs of spine instability or fusion/non-fusion were documented. The signs of instability in the follow-up CT were defined as the following: (1) the presence of radiolucent zones around the screws in combination with material loosening and loss of spinal stability; 2) pseudarthrosis in combination with progressive back pain. This combined approach of evaluating both clinical and imaging signs of instability are our institution’s decision-making criteria for failed spinal fusion. However, in cases of significant progressive back pain combined with the absence of strong signs of instability in CT scans (e.g., material loosening, radiolucent zones around pedicle screws, pseudarthrosis), further lateral lumbar flexion–extension X-ray images were reviewed. The evaluation of bone fusion was interpreted using the criteria described by Bridwell et al. [22].

### Statistical analysis

All data analysis was performed using IBM® SPSS® Statistics V22.0 (IBM, Chicago, Illinois, USA). Data on categorical variables are given as numbers and percentages. After normality testing via the Shapiro–Wilk test, continuous normally distributed data were compared using *t*-tests, while the Mann–Whitney *U* test was used for non-parametric data. Univariable analyses of nominal data among the defined groups were performed using Fisher’s exact test and a chi-squared test was used for multinomial data. Statistical tests resulting in a *p* value < 0.05 were considered as significant.

## Results

A total of 172 patients were surgically treated and included in the study. The majority of patients (54.0%) underwent lumbar spine surgery. Table 1 contains the baseline patient data. The median age was 66 years (range 57–74 years).

**Table 1:** Baseline patient data

Total	*n* = 172	%
**Sex**		
Female	57	34.0
Male	115	66.0
**Age, median (range) (years)**	66 (57–74)	
**ASA**		
ASA_1-2_	35	20.4
ASA_3-4_	137	79.6
**Single-level PSD**	114	66.3
**Multi-level PSD**	58	33.7
Distant	11	19.0
Adjacent	47	81.0
**Location**		
Cervical	13	7.6
Thoracic	31	18.0
Lumbar	93	54.0
Cervicothoracic junction	4	2.3
Thoracolumbar junction	14	8.1
Lumbo-sacral junction	17	10.0
**CCI, median (range)**	6.5 (3–9)	
**Early complications**	11	6.4
Surgery-related	5	
Adverse events	6	
**Material failure**	3	1.7
At 3 months	1	
At 12 months	2	
**Loss to follow-up at 12 month examination**	50	29.0

ASA: American Society of Anesthesiology score, CCI: Charlson comorbidity index, PSD: Pyogenic spondylodiscitis

Overall, 6.4% of the patients suffered from postoperative early complications. The median CCI score was 6.5 (range 3–9). Three patients (1.8%) suffered later material failure which needed revision.

Figure 1 is an MRI image showing single-level PSD (A) and adjacent multi-level PSD (B). Figure 2 is an MRI image showing multi-level PSD at the cervical und thoracic spine, A: Before surgery, B: After surgery.

In 19% (*n* = 11/58) of the patients with multi-level PSD the discitis was distant (Fig. 2 A), whereas in 81% (*n* = 47/58) of the patients the PSD levels were adjacent (Fig. 1 B). There were no significant differences in the rates of early fusion 3 months after surgery between the two groups (single-level and multi-level) or in the subgroup analysis of multi-level PSD (Tables 2 and 3). The loss to follow-up rate 12 months after surgery was 29.0%. Tables 4 and 5 show the late fusion outcome after 12 months. There is no significant difference between the single-level and multi-level groups or within the multi-level PSD subgroup.

**Table 2:** Early (3 months) fusion outcome in both groups

	*N*	Fusion	
All	172	Yes	No
Single-level	114	80	34
Multi-level	58	36	22
			*P* = 0.18*

*P*: *p* value. *Not significant

**Table 3:** Early (3 months) fusion outcome in multi-level subgroup

Multi-level	*N*	Fusion	
	58	Yes	No
Adjacent	47	30	17
Distant	11	6	5
			*P* = 0.27*

*P*: *p* value. *Not significant

**Table 4:** Late (12 months) fusion outcome in both groups

	*N*	Fusion	
All	122	Yes	No
Single-level	68	46	22
Multi-level	54	36	18
			*P* = 0.999*

*P*: *p* value. *Not significant

**Table 5:** Late (12 months) fusion outcome in multi-level subgroup

Multi-level	*N*	Fusion	
	54	Yes	No
Adjacent	45	30	15
Distant	9	6	3
			*P* = 0.73*

*P*: *p* value. *Not significant

The microbiological examination identified pathogens in 58.5% of the patients in the study cohort. The most common pathogenic bacterium (20%) was *Staphylococcus aureus*. A microbial infection was identified in the removed disc in 60% of cases, in the blood cultures in 30% of cases, and both in 10% of cases.

Table 6 shows univariable analyses using Fisher’s exact test (two-sided) and independent *t*-tests, which revealed no significant differences between the fusion and non-fusion groups.

**Table 6:** Univariable analysis after 12 months using Fisher’s exact test (two-sided) and independent *t*-test

Total (*n* = 122)	Fusion (*n* = 82)	Non-fusion (*n* = 40)	*p* value
**Sex**			0.10
Female (*n* = 40)	31 (77.5%)	9 (22.5%)	
Male (*n* = 82)	51 (62.2%)	31 (37.8%)	
**Age, median (range) (years)**	65 (57–74)	66 (57–78)	0.91
**ASA**			0.28
ASA_1-2_ (*n* = 34)	20 (58.8%)	14 (41.2%)	
ASA_3-4_ (*n* = 88)	62 (70.5%)	26 (29.5%)	
**Single-level PSD**	46	22	0.66
**Multi-level PSD**	36	18	0.99
Distant (*n* = 9)	6 (66.7%)	3 (33.3%)	
Adjacent (*n* = 45)	30 (66.7%)	15 (33.3%)	
**Location**			0.46
Cervical (*n* = 13)	10 (12.5%)	3 (7.5%)	
Thoracic (*n* = 20)	13 (15.6%)	7 (17.5%)	
Lumbar (*n* = 72)	52 (63.4%)	20 (50.0%)	
Other (*n* = 17)	7 (8.5%)	10 (25.0%)	
**CCI, median (range)**	5.5 (3–9)	7.5 (3–9)	0.88
**Material failure**	1	1	1.0

ASA: American Society of Anesthesiology score, CCI: Charlson comorbidity index, PSD: Pyogenic spondylodiscitis

A total of 23 patients were removed from the study who initially underwent surgery with suspected discitis. Here we had no evidence either intraoperatively from the aspect or later in the absence of evidence of germs as well as negative histopathological findings.

## Discussion

Data for this study were collected during routine clinical and radiological checks at our spine center at 3 and 12 months after urgent surgical treatment of pyogenic spondylodiscitis. The optimum time for routine follow-up examinations after spine surgery is strongly debated and various recommendations have been made. However, there is no high-level evidence identifying the precise point at which material fusion occurs after spine surgery [23].

The aim of this study was to evaluate the outcome of interbody fusion in combination with fixation for single-level and multi-level pyogenic spondylodiscitis. Despite most surgeons preferring surgical management of pyogenic spondylodiscitis, treatment recommendations are still a subject of controversy in the literature [24–27]. Pyogenic spondylodiscitis as a pathological condition is not uncommon. The clinical implications of this condition can be minor even in cases where imaging reveals typical signs of PSD. However, the clinical course can be also severe, with patients suffering from pain, neurological symptoms, and progressive deformity. The main goals of surgery are debridement with removal of the septic focus, collection of specimens for microbiological investigation, decompression of the spinal canal, stabilization, and bone fusion [28, 29]. Our study demonstrated that there is no significant difference between the fusion rates of multi-level (62.1%) and single-level (70.0%) PSD after treatment. Linhardt et al. reported the results of a prospective randomized trial, which investigated the long-term clinical and radiological outcomes after instrumented thoracic and lumbar fusions, comparing anterior–posterior to solely anterior instrumentation. This prospective trial included 22 patients with spondylodiscitis and also found high rates of fusion in both treatment arms [30].

Ackahota et al. analyzed the outcomes of multi-level vertebrectomy in spondylodiscitis. Twenty-two percent of the patients underwent revision surgery because of material dislocation or infection. The fusion rate was 78% in this study, which accords with our results [31]. Moreover, Lei He et al. also found a fusion rate of 80% after 1 year in a single center series with 31 patients undergoing minimally invasive surgery using the lateral transpsoas approach in combination with fusion [32]. The main pathophysiological mechanisms for implants loosening and non-fusion after spinal surgery are often considered to be mechanical causes [33], aseptic or low virulent implant-associated infections, as well as early implant infections [34].

The Lau et al. study group reported that smoking and nicotine consumption in general are strong risk factors for non-union after lumbar arthrodesis surgery because of possible tissue hypoxia and potential toxicity of osteoblasts [35]. We have not evaluated this factor in our study: 50 of our patients were lost to follow-up at 12 months, and information about nicotine consumption was missing for 20 other patients. However, we have included smoking status in the study limitations.

Our recommendation meets the criteria for a level of evidence 3, based on the publication by Kaiser et al. [36].

### Limitations

The major limitation of the present investigation is its retrospective nature in describing experience at a single institution. We were able to achieve an acceptable level of short-term follow-up but it is unclear to what extent the results for this small patient cohort allow us to recommend this treatment algorithm in all cases and in potential subgroups of high-risk patients. Additionally, we did not collect information about smoking status for all of our cohort. *In addition, the loss of patients in the follow-up of 29% leads to a limitation of the significance of the statistics and valuable informations is lost.*

## Conclusion

Surgical treatment of multi-level PSD is a safe option. Our study demonstrates that there was no significant difference between short-term fusion rates after surgical treatment of single-level or multi-level pyogenic spondylodiscitis. Furthermore, in the multi-level subgroup analysis, no significant differences in fusion outcome were observed between adjacent and distant PSD. Therefore, surgical therapy for multi-level PSD should not be omitted in this patient population.

## Data Availability

All data generated or analyzed during this study are included in this published article.

## Abbreviations

CCI: Charlson comorbidity index
CT: Computed tomography
ML: Multi-level
MRI: Magnetic resonance imaging
PSD: Pyogenic spondylodiscitis
PSIs: Patient safety indicators
SL: Single-level

## Code availability

'Not applicable' for that section.

## Declarations

### Study design

Retrospective clinical cohort study. The investigation was approved by the local ethics committee (reference no. 067/21).
